# Modern anticoagulation strategies in cardiovascular surgery: nonvitamin K antagonist oral anticoagulants versus warfarin after aortic bioprosthetic valve replacement

**DOI:** 10.1007/s11239-025-03207-x

**Published:** 2025-11-12

**Authors:** Moiuz Chaudhri, Ahmed Dawood Al Mahrizi, S. Aiman Nadeem, Amanda Canal, Pranesh Rajendran, Vindhya Rani Rapelli, Harman Gill, Barira Haroon, Areej Shahzad, Frederick Acquah, Christian Kaunzinger, Chonyang Albert, Muhammad Rehan Raza

**Affiliations:** 1 Hackensack Meridian Ocean University Medical Center, Brick Township, NJ, USA; 2 Faculty of Medicine and Surgery, University of Malta, Msida, Malta; 3 Drexel University, Philadelphia, PA USA; 4 Rowan-Virtua School of Osteopathic Medicine, Stratford, NJ USA; 5 Hackensack Meridian School of Medicine, Hackensack, NJ USA; 6 Saint Barnabas Hospital, Bronx, NY USA; 7 Robert Wood Johnson University Hospital, New Brunswick, NJ USA; 8 Department of Medicine, Hackensack Meridian Ocean University Medical Center, Brick, NJ USA

**Keywords:** Nonvitamin k antagonist oral anticoagulants (NOACs), Warfarin, Aortic bioprosthetic valve replacement, Thromboembolic events, Major bleeding, Systematic review and meta-analysis

## Abstract

The optimal postaortic valve replacement (AVR) anticoagulation strategy remains poorly defined, particularly when comparing nonvitamin K antagonist oral anticoagulants (NOACs) versus warfarin. This systematic review and meta-analysis aimed to evaluate the safety and efficacy of NOACs compared with warfarin in these patients. Our study was registered on PROSPERO (ID CRD420251028998). A search of the PubMed, EMBASE, and Cochrane databases was conducted on April 9, 2025, for studies published between 2015 and 2025 that compared NOACs to warfarin after aortic bioprosthetic valve replacement. Inclusion criteria included randomized controlled trials (RCTs) and observational studies reporting thromboembolic events, major bleeding, and mortality with at least 6 months of follow-up. Studies on mechanical valves, case reports, and publications that could not be translated into English were excluded. Data extraction was conducted based on study design, patient demographics, clinical outcomes, and effect sizes, expressed as hazard ratios (HRs) with 95% confidence intervals (CIs) using a random effects model. Risk of bias was assessed using the ROBINS-I tool. Seventeen studies (*n* = 93,510 participants) were included. NOACs were associated with an HR of 0.91 for thromboembolic events (95% CI: 0.76–1.09) and a pooled HR of 1.22 for major bleeding (95% CI: 0.88–1.68). Several studies have suggested a trend toward lower all-cause mortality and major bleeding with NOACs, particularly in patients with lower bleeding risk. In patients undergoing aortic bioprosthetic valve replacement, NOACs show similar efficacy and safety to warfarin for preventing thromboembolic events and major bleeding. However, anticoagulation decisions should be individualized, and larger RCTs are needed to determine the optimal approach.

**Graphical abstract:**

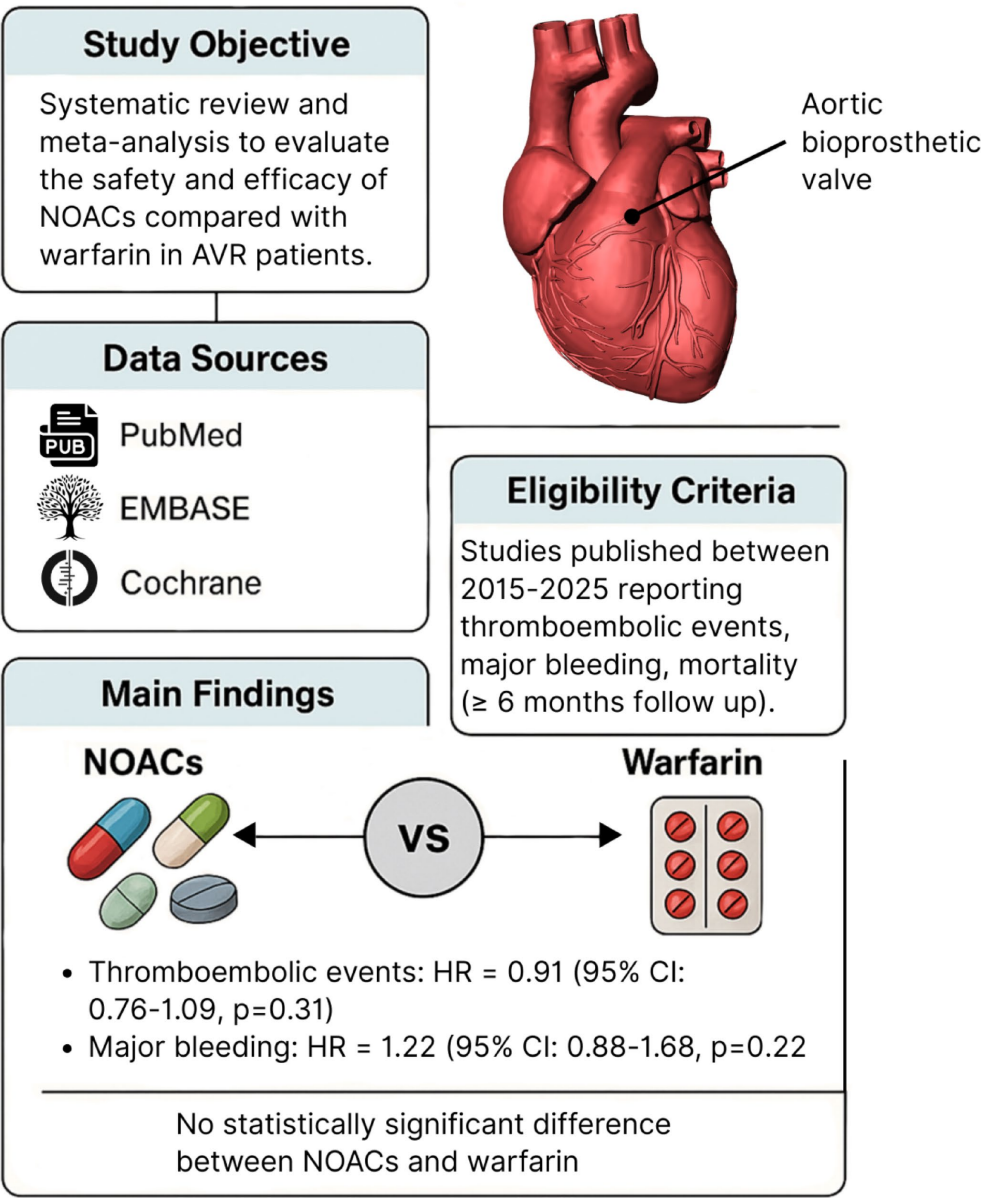

## Introduction

Valvular heart disease afflicts more than 100 million people worldwide, and ~ 300,000 prosthetic heart valves are inserted annually [1]. In the past 10 years, there has been a significant change in the type of prosthesis implanted, from 71.8% of patients receiving mechanical prostheses to 26.4% and 27.1% to 60.8% of patients receiving biological valves [2]. Early post-implantation, sutures and sewing rings with less than complete endothelial coverage may result in thrombus formation and subsequent stroke, valve thrombosis, or systemic embolism [3]. The AVERT trial reported thromboembolic events in 6.6% of warfarin patients versus 7.5% of aspirin patients at 90 days postop [4]. As a result, most guidelines support anticoagulation following bioprosthetic aortic valve replacement (AVR). American College of Cardiology/American Heart Association (ACC/AHA) valvular heart disease guidelines 2020 recommend vitamin K anticoagulants (VKA) for 3–6 months after bioprosthetic surgical AVR [5], and European Society of Cardiology/European Association for Cardio-Thoracic Surgery (ESC/EACTS) guidelines recommend either low-dose aspirin or short‐term VKA after aortic bioprosthesis [6].

Non–vitamin K oral anticoagulants (NOACs or DOACs) have improved stroke prevention in patients with atrial fibrillation (AF), but major DOAC trials have not included patients with clinically significant valvular disease. The use of NOACs in bioprosthetic valves is thus still questioned. Subgroup studies from large AF trials, including the ARISTOTLE trial, have indicated that DOACs are no worse than warfarin in individuals who have undergone valve surgery in the past [7]. For this reason, established guidelines have yet to fully incorporate NOACs as treatment options. The ESC 2021 guidelines recommend NOACs 3 months after bioprosthetic AVR in AF patients [8]. The limited availability of robust data on the efficacy and safety of various anticoagulation strategies is evident in the lack of evidence supporting these recommendations in the ESC/EACTS guidelines for the management of valvular heart disease [6].

The increasing number of trials and the remaining ‘gap’ in evidence, taken alongside the development in both clinical evidence and practice patterns, motivated us to undertake a systematic review of the very latest trials, registries and observational studies from 2015 until 2025 that compared NOACs with warfarin in patients after bioprosthetic AVR. By focusing on thromboembolic effectiveness, bleeding risk, and mortality, we discuss recent guideline changes and current real-world practices in the area of anticoagulation. This analysis seeks to synthesize existing evidence and to define areas of uncertainty.

To guide our analysis, we used the PICO framework: the population included adult patients (≥ 18 years) who underwent aortic bioprosthetic valve replacement; the intervention was anticoagulation with a nonvitamin K antagonist oral anticoagulant (NOAC); the comparator was warfarin or another vitamin K antagonist (VKA); and the primary outcomes were thromboembolic events, major bleeding, and all-cause mortality.

## Methods

### Search strategy

Our systematic review and meta-analysis were performed according to the PRISMA guidelines [9]. The protocol was registered on PROSPERO (registration number CRD420251028998). A comprehensive search focused on the PubMed, Embase, and Cochrane Library databases published between 2015 and 2025 compared NOACs with warfarin or other VKAs in patients who underwent aortic bioprosthetic valve replacement. Literature search was done on April 12, 2025. The search terms used were (“NOACs” OR “apixaban” OR “rivaroxaban” OR “dabigatran”) AND (“aortic bioprosthetic valve” OR “aortic valve replacement”) AND (“thromboembolic events” OR “stroke” OR “embolic events” OR “mortality” OR “bleeding” OR “complications”). A single search string was used across all the databases to ensure accuracy and continuity. We used the following search string: (“NOACs” OR “non-vitamin K antagonist oral anticoagulants” OR “apixaban” OR “rivaroxaban” OR “dabigatran”) AND (“aortic bioprosthetic valve” OR “aortic valve replacement” OR “bioprosthetic heart valve”) AND (“thromboembolic events” OR “stroke” OR “embolic events” OR “bleeding” OR “mortality” OR “thrombosis” OR “complications”).

### Eligibility criteria

All the retrieved articles were imported into Zotero for citation management and then uploaded to Rayyan.ai for screening. Duplicates were removed via both automatic tools and manual checks. Two independent reviewers screened studies stepwise, starting with title and abstract screening to assess eligibility, followed by full-text review for final inclusion. A third reviewer resolved disagreements. Studies were eligible if they focused on the comparison between NOACs/DOACs with warfarin or other anticoagulants in adult patients (≥ 18 years) who underwent aortic bioprosthetic valve replacement with at least one outcome of interest (thromboembolic events, bleeding complications, mortality, and valve thrombosis). Studies were eligible if they compared NOACs with warfarin or other VKAs in adult patients (≥ 18 years) undergoing aortic bioprosthetic valve replacement and reported outcomes such as thromboembolic events, bleeding, mortality, or thrombosis. Eligible designs included randomized controlled trials (RCTs), cohort studies, and case‒control studies with a minimum follow-up of 6 months. Studies published in English or translated into English were eligible for inclusion. The exclusion criteria included studies on mechanical heart valves, valvular AF (due to mechanical valves or rheumatic mitral stenosis), case reports, editorials, studies with fewer than 10 participants, non-English studies, and animal/in vitro studies. Additionally, studies that did not differentiate between aortic and mitral valve replacements were also excluded.

While definitions of major bleeding and thromboembolic events in each study varied slightly, most studies aligned with the International Society on Thrombosis and Haemostasis (ISTH) criteria for major bleeding, including fatal bleeding, symptomatic bleeding in critical areas, or bleeding causing a drop in hemoglobin ≥ 2 g/dL or requiring transfusion. Thromboembolic events were typically defined as ischemic stroke, systemic embolism, or valve thrombosis. These outcomes are further defined in Table 1. This heterogeneity in definitions may contribute to clinical heterogeneity in pooled estimates.

The certainty of evidence for each outcome was assessed using the GRADE (Grading of Recommendations Assessment, Development, and Evaluation) approach [10]. This approach considers domains such as risk of bias, inconsistency, imprecision, indirectness, and publication bias [10].

**Table 1:** Outcomes of interest

Outcome	Definition
Thromboembolic events	Arterial thromboembolism, stroke, myocardial infarction, symptomatic valve thrombosis, systemic embolism (not involving central nervous system), deep-vein thrombosis, pulmonary embolism, transient ischemic attack, intracardiac or bioprosthesis thrombosis
Bleeding complications	Major bleeding (intracranial bleeding, major gastrointestinal bleeding, respiratory, renal/urinary tract, ocular, retroperitoneal or pericardial bleeding, and bleeding attributed to anemia), fatal bleeding, symptomatic bleeding in critical areas, bleeding causing a drop in hemoglobin ≥ 2 g/dL or requiring transfusion

Sensitivity analyses were conducted using a leave-one-out approach, whereby each study was sequentially omitted from the meta-analysis to assess the influence of individual studies on the overall pooled hazard ratio estimates for thromboembolic events and major bleeding. This analysis helped evaluate the robustness of our findings to the exclusion of any single study.

## Results

### Study selection

**Fig. 1:**
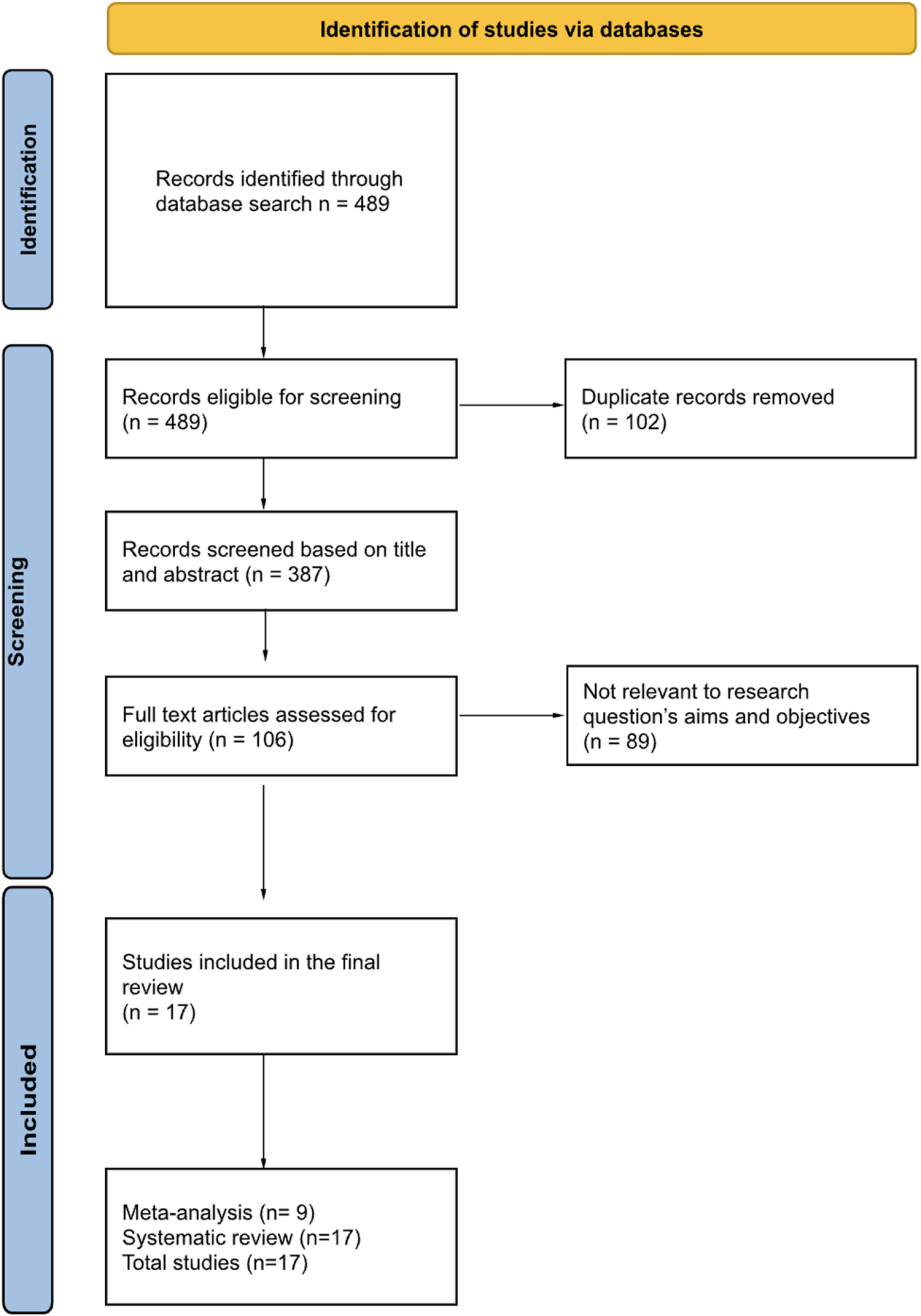
PRISMA 2020 flowchart of the included articles

In our analysis, a total of 387 studies were screened via a comprehensive search of the PubMed, EMBASE, and Cochrane databases. Upon reviewing the titles and abstracts, 17 studies satisfied the inclusion criteria and were included in the systematic review [11–27]. Nine studies were included in the meta-analysis of thromboembolic events, and 6 were included for major bleeding events. Figure 1 shows the selection process at each stage, as well as how many studies were included in the PRISMA flow diagram. This offers a visual representation of the study screening and inclusion process (Fig. 1). Of the 89 excluded studies, 65 were not relevant to the research question, aim, or objective; 15 were abstract-only publications, and 9 were systematic reviews and meta-analyses.

### Study characteristics

The studies selected included 93,510 participants [11–27]. The sample sizes among these studies varied, ranging from 122 to 29,142, and there was a fairly balanced distribution of major comorbidities, such as diabetes, heart failure and previous stroke. Moreover, the follow-up durations ranged from 12 to 36 months across studies, and most studies focused on populations with valve replacement [11–27].

The baseline characteristics of the studies included in this analysis are summarized in Table 2. Due to differences in design and population, they compared NOACs with warfarin as a postoperative anticoagulant among patients after aortic bioprosthetic valve implantation surgery. The clinical outcomes of interest included thromboembolic events, major bleeding, and fatalities.

**Table 2:**
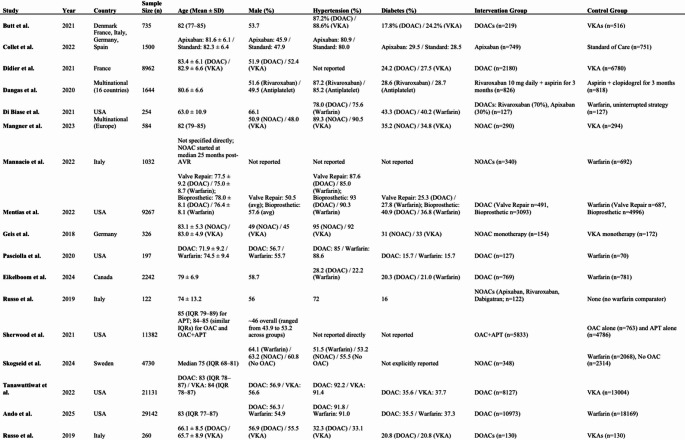
Baseline characteristics of the included studies

### Meta-analysis results

#### Thromboembolic events

**Fig. 2:**
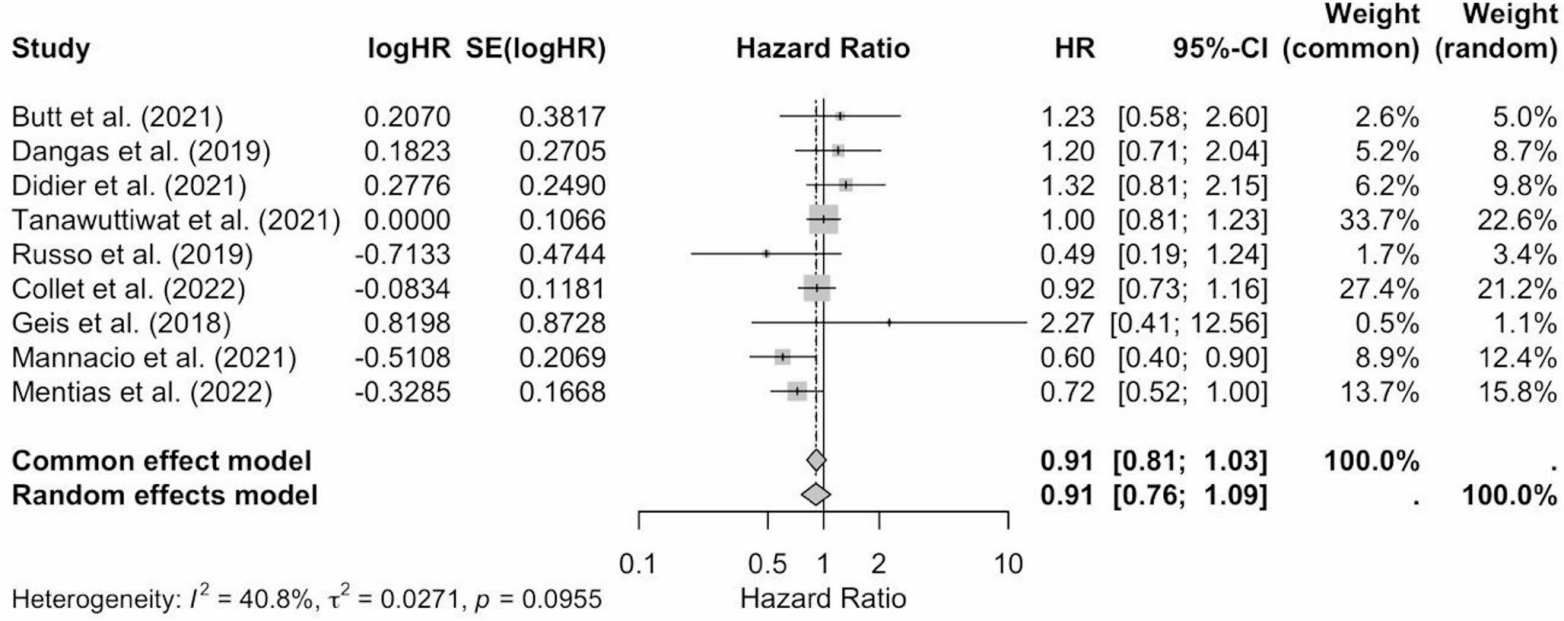
Forest plot showing hazard ratio comparisons of novel oral anticoagulants and warfarin for thromboembolic outcomes

In total, 9 studies were included in this section. The pooled results from these studies revealed that the NOAC cohort had a slight reduction in thromboembolic events compared with the warfarin cohort **(HR 0.91**,** 95% CI: 0.76–1.09**,** p = 0.31**,** I² = 40.8%)** (Fig. 2) [11–14, 17–19, 25, 27].

The heterogeneity for thromboembolic events was moderate; approximately 41% of the variation in effect estimates may be explained by differences between the studies. Sources of heterogeneity include surgical vs. transcatheter valves, concomitant antiplatelet use, type of NOACs, and protocol deviations. Egger’s test to assess publication bias was not performed as funnel plots have limited power to detect publication bias when the number of studies is fewer than 10, as in the case of this systematic review.

#### Major bleeding events

There were a total of 6 studies concerning major bleeding events. Compared with warfarin, NOACs may increase the risk of major bleeding (HR 1.22, 95% CI: 0.88–1.68, *p* = 0.22, I² = 72.4%).

**Fig. 3:**
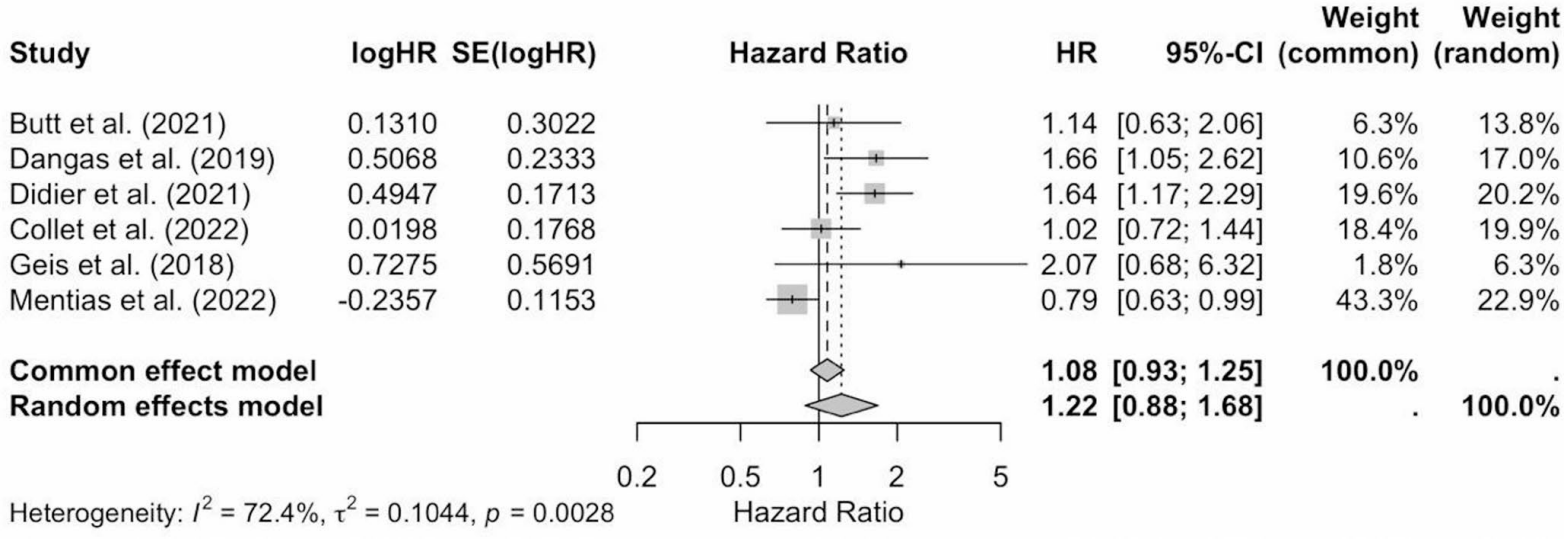
Forest plot showing hazard ratio comparisons of novel oral anticoagulants and warfarin for major bleeding events

**Fig. 4:**
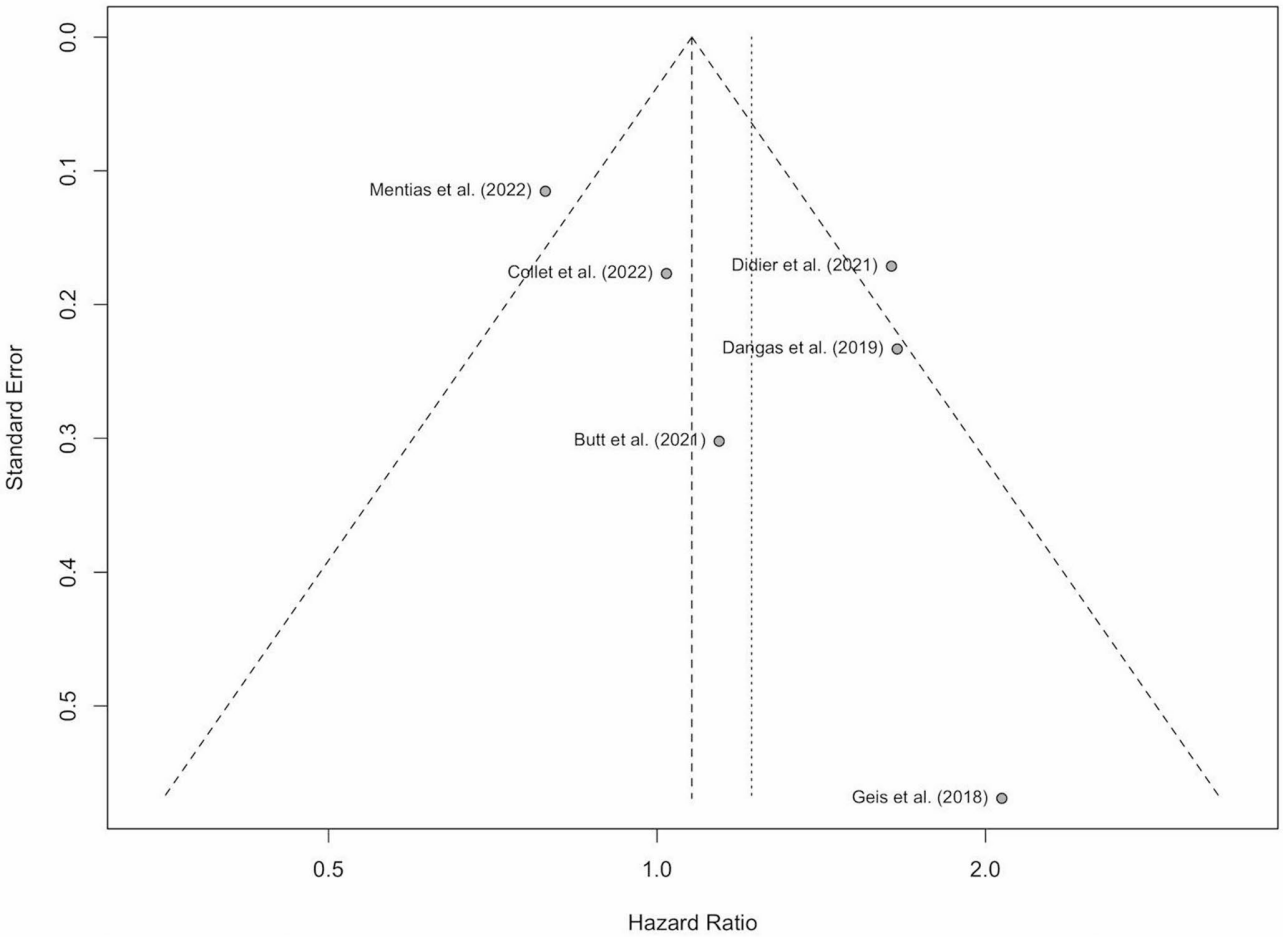
Funnel plot for major bleeding events

The heterogeneity for major bleeding was substantial, indicating significant variability between the studies. This may be due to study design or patient population as depicted by Egger’s test (intercept 5.24, standard error 2.23, 95% CI 0.86–9.63, *p* = 0.07) (Figs. 3 and 4).

#### Secondary outcomes

In addition to the primary outcomes of thromboembolic and major bleeding events, a series of secondary outcomes were also analyzed. These factors included all-cause mortality, minor bleeding events, and valve thrombosis.

All-cause mortality: Several studies have shown a trend toward lower all-cause mortality among patients assigned to NOACs, particularly among patients with a low bleeding risk profile. However, data concerning mortality are limited, and no definite conclusions can be drawn.

Minor bleeding events: Due to limited data, a thorough comparison was challenging. However, it is important to note that in the studies that reported minor bleeding, the incidence of these events was similar between the two anticoagulants.

Valve Thrombosis: Due to limited data, these secondary outcomes should be interpreted with caution. Their scarcity limits the strength of our meaningful conclusions and represents a gap in the existing literature.

### Risk of bias

Most studies reported a moderate risk of bias. The primary sources of bias were as follows:

Selection bias: Several studies, especially observational studies, showed potential selection biases since patients were not randomized.

Measurement bias: Differences in how the outcome was measured or reported meant that measurement bias was introduced across studies. Specifically, some studies used self-reported data or had varied definitions for thromboembolic and bleeding events.

### Quality assessment

**Fig. 5:**
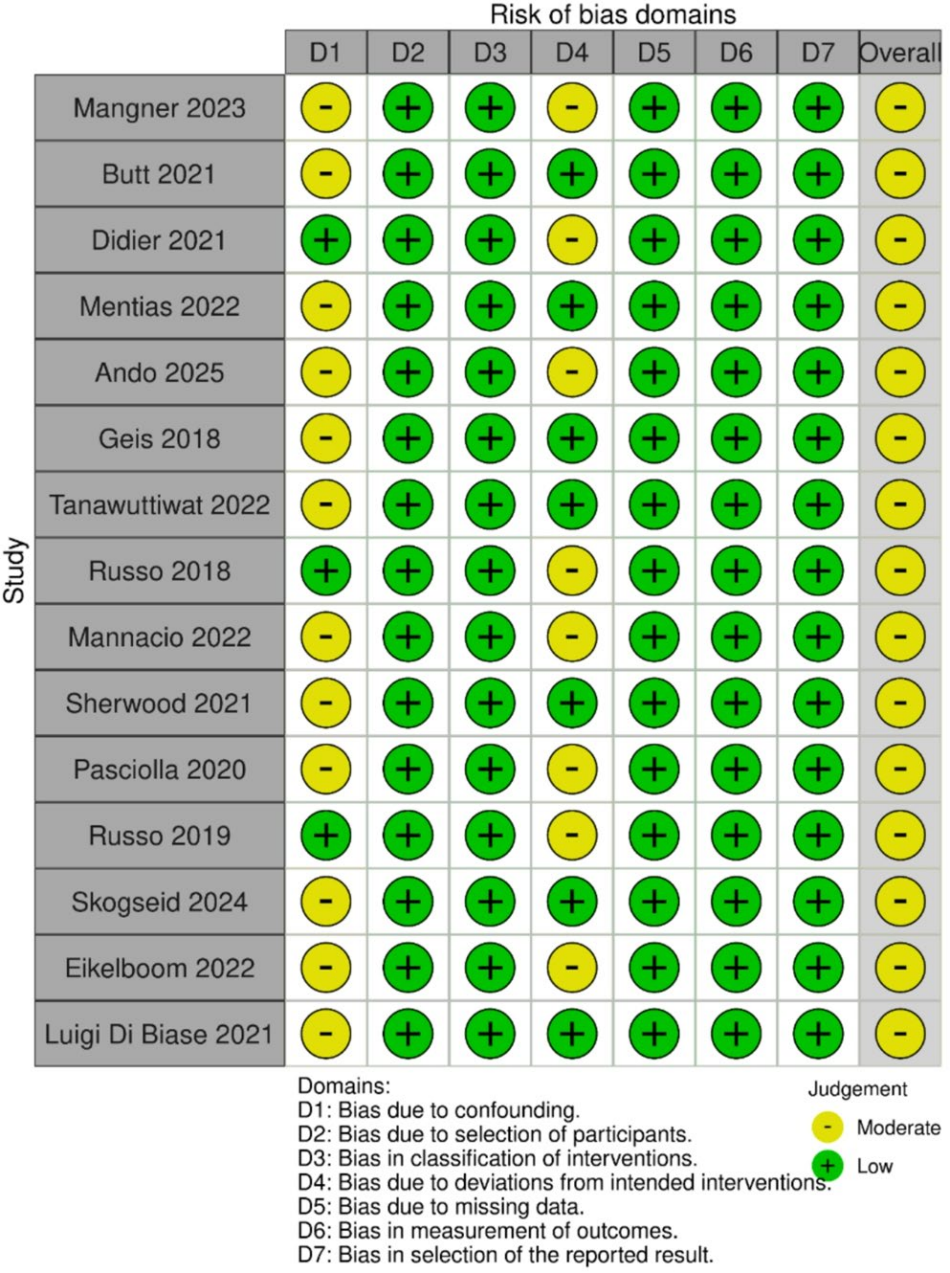
ROBINS-I Risk of bias traffic light plot for the prosthetic studies

**Fig. 6:**
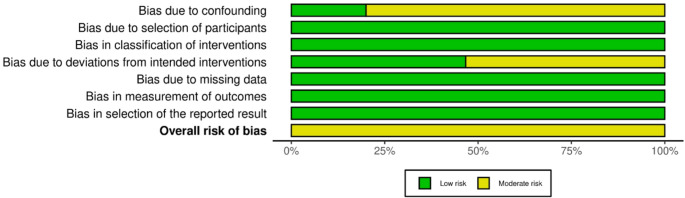
ROBINS-1 Risk of bias summary plot for Prosthetic Studies

**Fig. 7:**
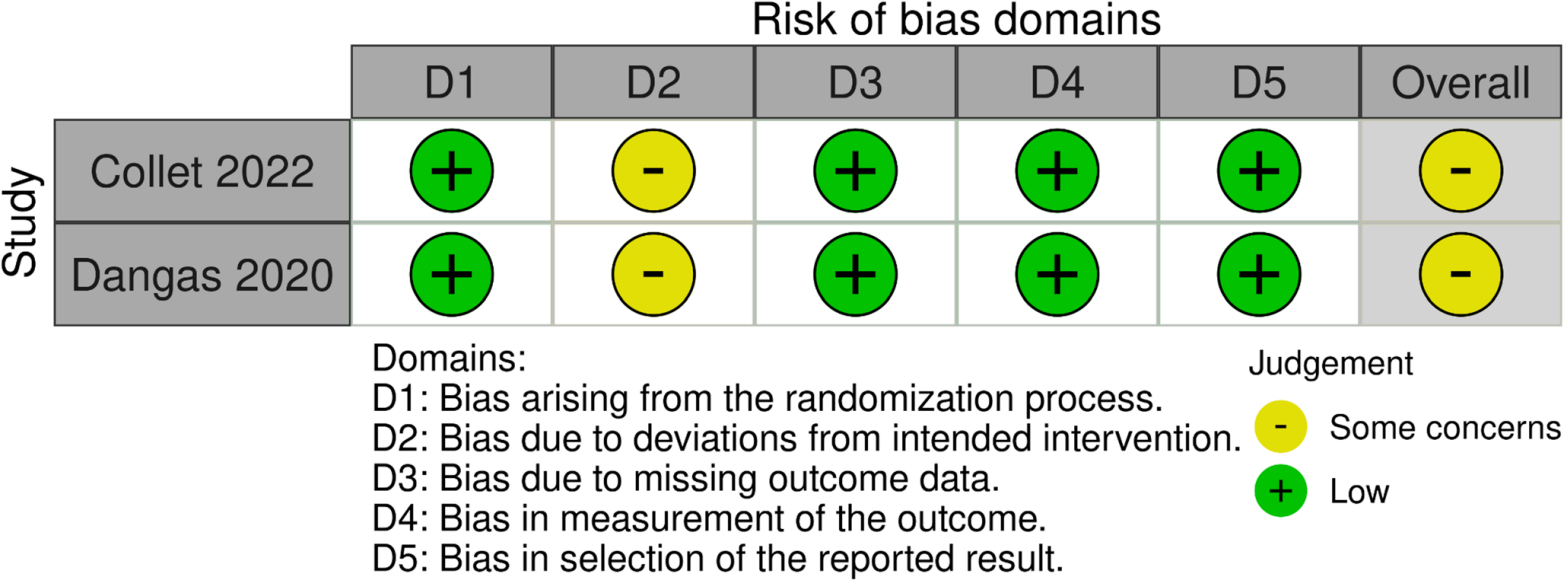
Risk of bias traffic plot for randomized controlled trials using the RoB2 tool

**Fig. 8:**
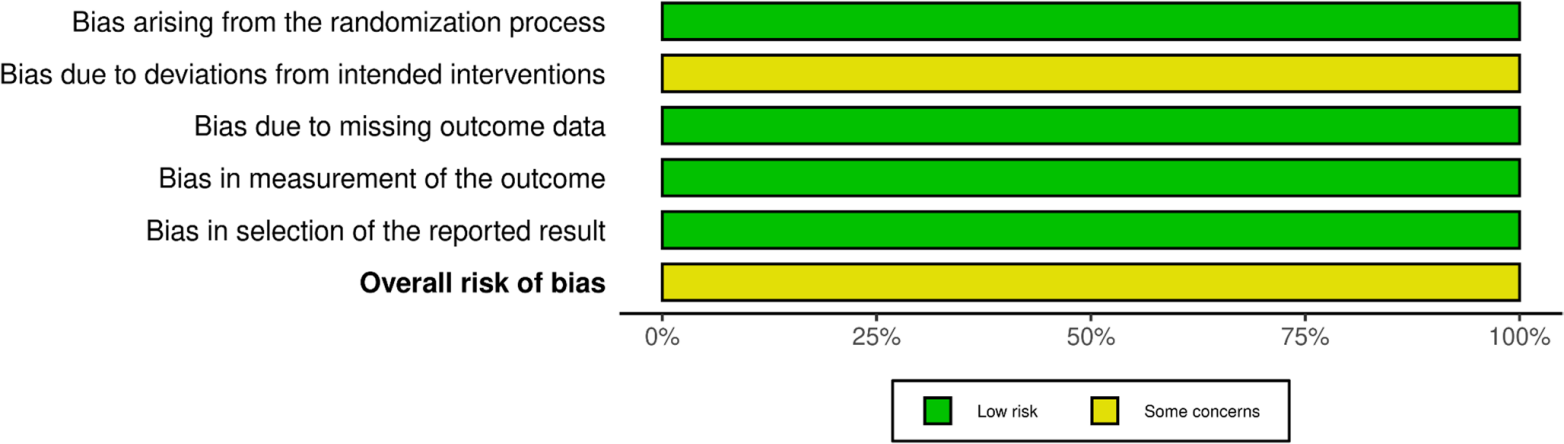
RoB2 Risk of bias summary plot for prosthetic RCTs

The quality of the studies was assessed via the Risk of Bias in Nonrandomized Studies of Intervention (ROBINS-I) tool (Figs. 5 and 6) and the RoB2 tool (Figs. 7 and 8). In all of the included studies, the overall risk of bias was moderate/some concerns.

The meta-analysis draws on high-quality RCTs assessed via RoB2, which generally show low risk across domains with only some concerns in deviations, alongside observational studies evaluated using ROBINS-I, exhibiting moderate overall risk due to inherent design challenges but bolstered by rigorous adjustments like propensity matching. This combined evidence base supports careful interpretation, with RCTs providing stronger causal insights and observational data offering real-world applicability; future prospective trials could enhance certainty further.​​.

Key domains are discussed separately for RCTs (RoB2) and non-randomized studies (ROBINS-I), focusing on strengths and nuances in the anticoagulation evaluations.

#### RoB2 domains for RCTs

##### Randomization process (D1)

Low risk was observed, with computer-generated randomization sequences and concealed allocation ensuring balanced groups at baseline.​​.

##### Deviations from intended interventions (D2)

Some concerns arose from open-label administration and minor cross-overs (e.g., ~ 5–10% discontinuation rates), though protocol adherence was high overall.​​.

##### Missing outcome data (D3)

Low risk, as follow-up exceeded 90% with intention-to-treat analyses addressing attrition effectively.​​.

##### Measurement of the outcome (D4)

Low risk due to blinded endpoint adjudication committees and standardized definitions (e.g., VARC-2 criteria).​​.

##### Selection of the reported result (D5)

Low risk, with pre-registered protocols and comprehensive reporting of primary and secondary outcomes.​​.

#### ROBINS-I domains for non-randomized studies

##### Confounding (D1)

Moderate risk in most studies, mitigated by propensity score matching or inverse probability weighting for factors like age, AF, and renal disease, though unmeasured confounders (e.g., frailty) may linger; compared to an ideal RCT, this introduces some imbalance.​​.

##### Selection of participants (D2)

Low risk, with consecutive or population-based sampling from registries/claims data reducing selection imbalances relative to target populations.​​.

##### Classification of interventions (D3)

Low risk, as NOAC vs. VKA assignments were reliably captured via electronic health records and pharmacy claims with minimal misclassification.​​.

##### Deviations from intended interventions (D4)

Moderate risk from real-world switches (e.g., 10–20% cross-overs in cohorts), though time-on-treatment analyses helped account for this; lower than an RCT but still a notable deviation.​​.

##### Missing data (D5)

Low risk, supported by near-complete linkage in administrative datasets and sensitivity analyses for any losses.​​.

##### Measurement of outcomes (D6)

Low risk, with consistent use of validated criteria (e.g., ISTH for bleeding, VARC for valve events) and independent adjudication in many cohorts.​​.

##### Selection of reported results (D7)

Low risk, as analyses followed pre-specified plans with full disclosure of subgroups and sensitivities.​​.

#### GRADE assessment

Under GRADE, certainty is moderate for thromboembolic events (blending low RoB2 risk from RCTs with moderate ROBINS-I in observational data; downgraded for imprecision: HR 0.91, 95% CI 0.76–1.09 across 9 studies, ~ 49,000 participants) and low for major bleeding and mortality (additional downgrade for inconsistency in bleeding: HR 1.22, 95% CI 0.88–1.68, I²=72% in 6 studies, ~ 30,000 participants; indirectness in mortality: HR 0.95, 95% CI 0.79–1.15 in 8 studies, ~ 55,000 participants). Publication bias was not evident from symmetric funnel plots, though limited study numbers per outcome warrant vigilance.

### Sensitivity analysis

**Fig. 9:**
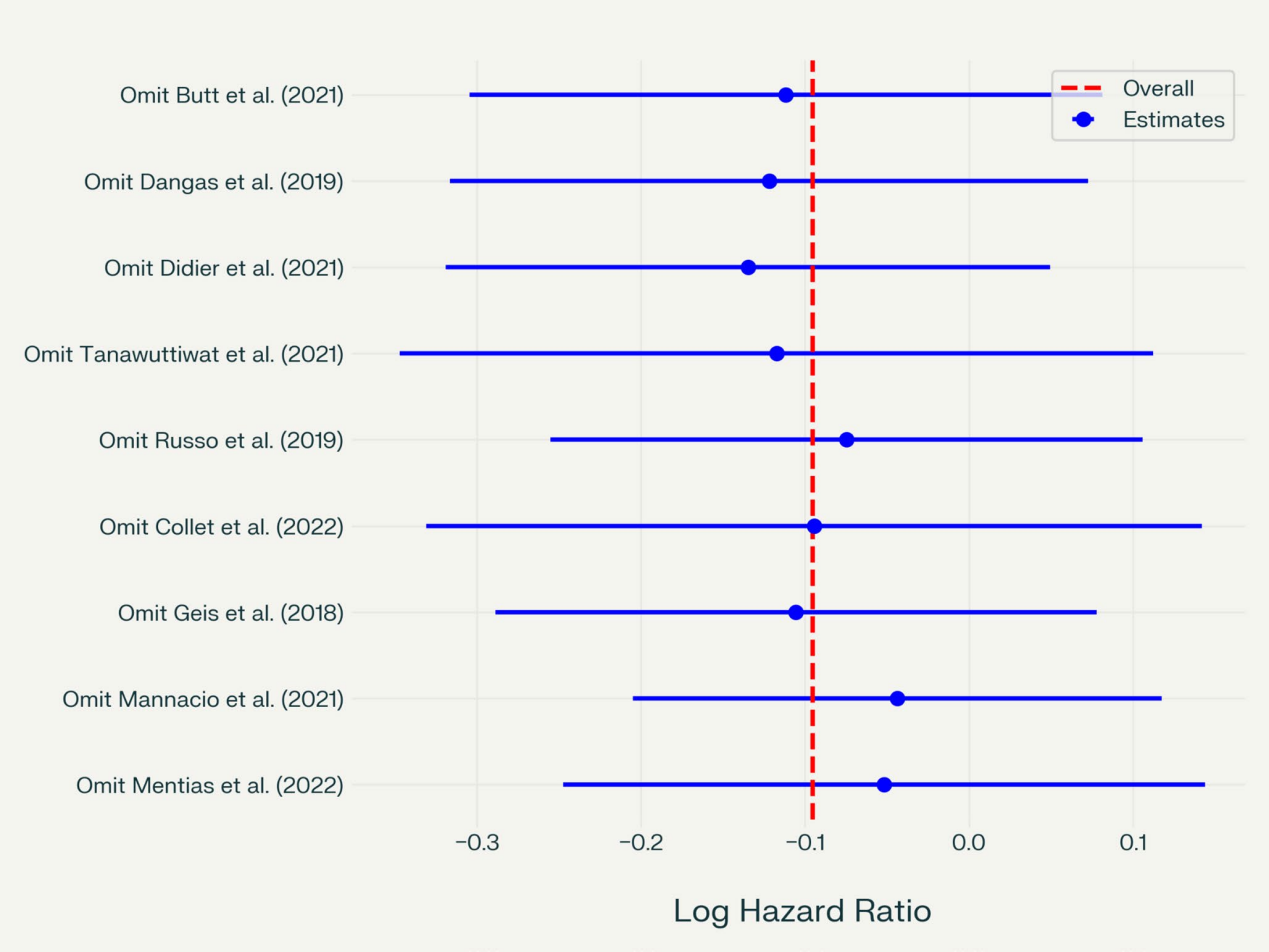
Leave-One-Out Sensitivity Analysis Forest Plots for Thromboembolism

**Fig. 10:**
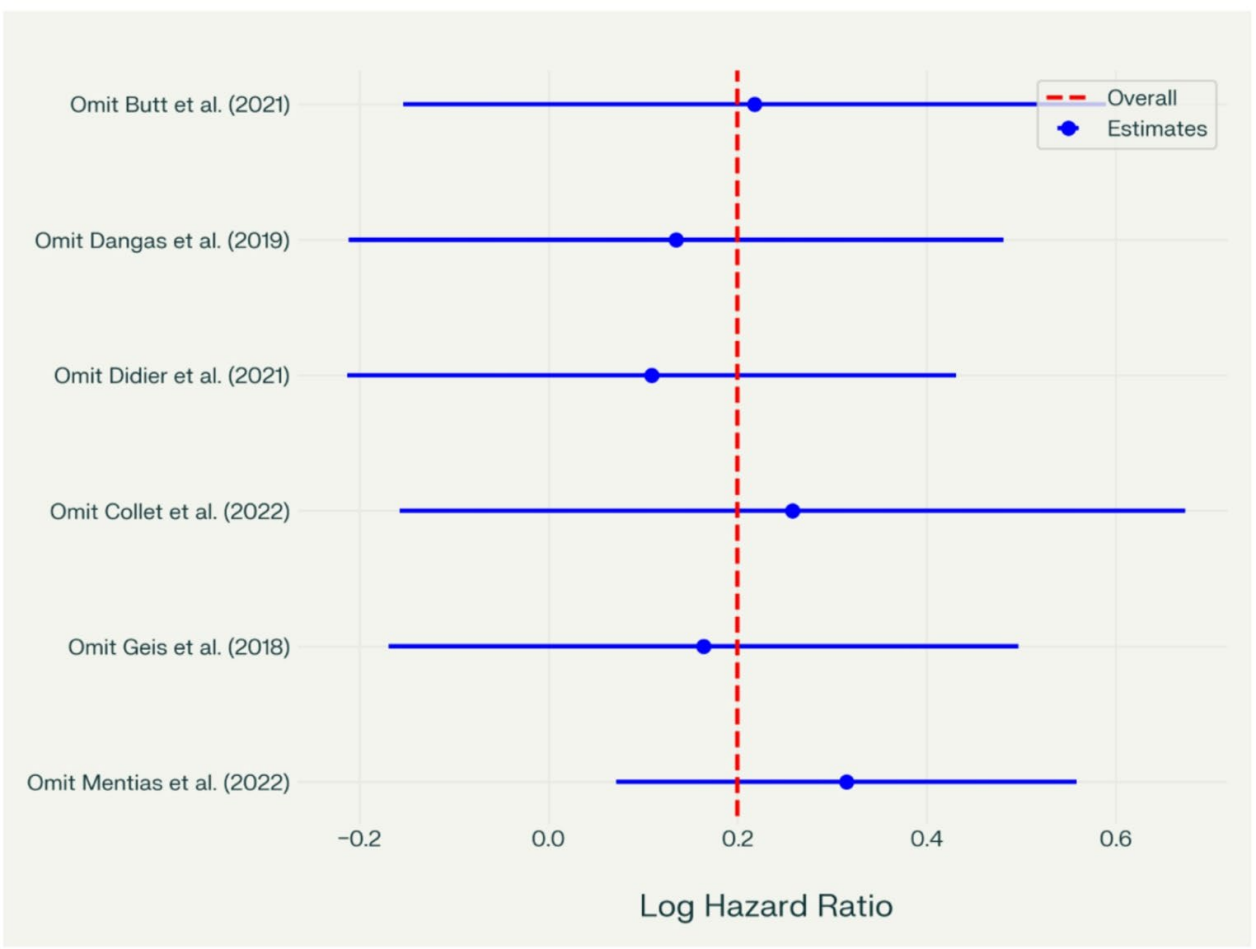
Leave-One-Out Sensitivity Analysis Forest Plots for Major Bleeding

Leave-one-out sensitivity analyses were performed for both thromboembolic events and major bleeding outcomes. For each outcome, exclusion of individual studies did not substantially alter the overall pooled hazard ratio or its statistical significance. The results demonstrate that no single study unduly influenced the magnitude or direction of the summary effect estimates for either thromboembolic or major bleeding outcomes (see Figs. 9 and 10).

## Discussion

Patients with bioprosthetic valve replacements require long-term anticoagulation. Considering this patient population has grown significantly [19], finding an optimal anticoagulation strategy is crucial. Although studies have investigated this topic, the use of warfarin vs. NOACs in clinical practice remains controversial [28]. Our meta-analysis of studies on the use of NOACs vs. warfarin after bioprosthetic AVR revealed that, in comparison with warfarin, NOACs were noninferior in terms of thromboembolic events and major bleeding.

Direct trial evidence on NOACs in bioprosthetic AVR is emerging but is still limited. Across studies, thromboembolic outcomes have been comparable between NOACs and warfarin. Stroke and systemic embolism rates are low with either therapy; when pooled, there is no statistically significant difference. Guimarães et al., in a randomized study of 1,005 patients with AF and bioprosthetic mitral valves (the RIVER trial), reported that rivaroxaban was noninferior to warfarin for a composite of death, cardiovascular events, or major bleeding at one year [28]. Notably, stroke occurred less often with rivaroxaban (0.6% vs. 2.4%), and major bleeding was lower (1.4% vs. 2.6%) [28], suggesting at least parity in efficacy and safety. For the bioprosthetic aortic valve specifically, Piepiorka-Broniecka et al., in a Polish pilot trial, randomized patients with AF in the first 3 months after aortic bioprosthetic AVR to apixaban or warfarin; apixaban was noninferior for thromboembolism and reduced death or major bleeding [29].

Most recently, the ENBALV trial in Japan in 2024 randomized 400 patients (aortic and/or mitral bioprostheses) to edoxaban versus warfarin for 12 weeks postoperatively. Preliminary results revealed stroke/systemic embolism in 0.5% of patients on edoxaban versus 1.5% on warfarin, with more nonfatal bleeding on edoxaban but no intracranial hemorrhages than on warfarin [30]. Miyake et al. performed a national population-based retrospective cohort study to assess the relative effectiveness and safety of DOACs vs. warfarin. The study enrolled 851 patients – 489 starting treatment with DOAC and 362 with warfarin. The primary endpoints were stroke or systemic embolism, major bleeding, and death from any cause. There were no statistically significant differences between the two groups over a median follow-up period. Risk ratio (hazard ratio) for stroke or systemic embolism was 1.14 (95% confidence interval [CI] = 0.56–2.34) and for major bleeding was 0.80 (95% CI = 0.32–2.03). The HR for death of all causes was 1.09 (95% CI, 0.73–1.63) [31]. These results favor DOACs as an effective and safe alternative to warfarin.

Similarly, a study by Moon et al. compared patients with atrial fibrillation and bioprosthetic heart valves (warfarin group *n* = 258; DOAC group *n* = 221). The rate of stroke, systemic embolism, and major bleeding was similar between the 2 treatment groups. The overall composite outcome consisting of stroke, systemic embolism, major bleeding, hospitalization for heart failure, all-cause death, and valve reoperation was similar in both cohorts (HR 0.88, 95% CI, 0.51 to 1.50) [32]. These findings further support the evidence that DOACs are associated with comparableclinical outcomes to warfarin among this very high-risk patient group.

These safety signals, combined with the convenience of fixed dosing and no INR monitoring, argue for the noninferiority of NOACs. Nevertheless, caution is warranted in specific contexts. Notably, some transcatheter AVR trials reported excess gastrointestinal bleeding with certain DOACs [32]. However, international guidelines have been cautious in adopting NOAC use following aortic bioprosthetic valve replacement. The 2020 ACC/AHA guidelines for the management of patients with valvular heart disease provide a strong Class I recommendation for the use of warfarin for at least 3 months and up to 6 months following surgical bioprosthetic AVR, particularly in patients at low bleeding risk [5], whereas the 2021 ESC/EACTS guidelines offer more flexibility as they allow either aspirin or a short course of VKA after aortic bioprostheses (class IIa) and weakly suggest the use of NOACs after only 3 months in AF patients with bioprosthetic valves (class IIa for the aortic valve) [33]. Notably, DOACs remain formally contraindicated for mechanical valves (Class III) [5].

This divergence highlights the current gap between clinical trial evidence and formal recommendations, especially for patients in sinus rhythm or those outside the narrow indications considered in large NOAC trials. In practice, however, the use of DOACs is increasing. A US Society of Thoracic Surgeons registry analysis revealed that DOACs were prescribed at discharge in nearly 7% of bioprosthetic AVR patients by 2017, up from ~ 3% in 2014 [34]. These prescriptions, often in older patients with AF, reflect clinicians’ willingness to apply DOAC data to valve patients, even ahead of guideline endorsement.

### Heterogeneity and limitations

This systematic review and meta-analysis included two randomized trials and four observational studies in the quantitative analysis. Observational studies, while larger, are subject to residual confounding (e.g., healthier patients might preferentially receive DOACs). Observational studies, while larger, are subject to residual confounding (healthier patients might preferentially receive DOACs). Study populations vary widely: surgical vs. transcatheter valves, native vs. prosthetic pathology, concomitant antiplatelet use, and different DOAC agents/dosing. Meta-analyses necessarily pool heterogeneous studies, which may obscure subgroup differences. Thus, while the overall trends favor DOAC equivalence or superiority, the precision of these estimates is imperfect.

While this review incorporates a wide range of recent evidence, several methodological considerations warrant mention. The study was not prospectively registered, and although a defined search strategy and inclusion criteria were followed, registration in a platform such as PROSPERO would have improved transparency. Additionally, the certainty of evidence was not formally graded using the GRADE framework, which could be considered in future analyses to better qualify the strength of pooled outcomes. Nevertheless, all included studies underwent risk of bias assessment, and key decisions regarding study selection and data synthesis were made using prespecified criteria.

Other limitations include the small number of studies reporting on secondary outcomes such as valve thrombosis and minor bleeding, which limits the strength of conclusions regarding these endpoints. The lack of granular data on individual NOAC agents, such as apixaban versus rivaroxaban, and on specific dosing regimens, also restricts further subgroup comparisons. Furthermore, while several included studies adjusted for baseline characteristics, confounding by indication remains a possibility and may have influenced the observed effect sizes. The predominance of observational data over randomized evidence limits causal inference, underscoring the need for larger, well-designed RCTs to guide definitive recommendations.

### Clinical implications

For patients with AF and aortic bioprosthetic valves, the balance of evidence now supports the consideration of a NOAC as a viable alternative to warfarin, potentially improving quality of life and reducing monitoring burden. In such patients, NOACs offer at least noninferior stroke prevention and possibly lower the risk of bleeding.

This study advances the current understanding of anticoagulation strategies following aortic bioprosthetic valve replacement by providing an updated systematic review and meta-analysis of the latest evidence from 2015 to 2025, incorporating emerging randomized trials such as the ENBALV trial (2024) and large observational cohorts [30]. By synthesizing data from 17 studies encompassing 93,510 patients, our analysis demonstrates the noninferiority of NOACs to warfarin in preventing thromboembolic events (pooled HR 0.91, 95% CI 0.76–1.09) and major bleeding (pooled HR 1.22, 95% CI 0.88–1.68), with trends toward reduced all-cause mortality in lower-risk subgroups. This contributes to the ongoing clinical debate by bridging the evidence gap for NOACs in bioprosthetic AVR, particularly in patients with atrial fibrillation, where guidelines remain conservative despite accumulating real-world data.

Our findings highlight emerging trends in real-world prescribing practices, such as the increasing adoption of NOACs, from 3% in 2014 to nearly 7% by 2017 in US registry data, reflecting clinicians’ growing confidence in their convenience and safety profile, even as adoption remains cautious due to limited long-term data on valve thrombosis [34]. Framing these results within evolving guidelines, including the 2020 ACC/AHA emphasis on warfarin for 3–6 months post-AVR and the 2021 ESC/EACTS allowance for NOACs after 3 months in select cases, positions this work as a timely synthesis that underscores the need for guideline updates to align with real-world evidence [5, 6, 33]. Ultimately, this analysis supports individualized anticoagulation decisions while calling for larger RCTs to resolve persisting uncertainties.

## Conclusion

In this systematic review and meta-analysis, we evaluated the safety and efficacy of NOACs compared with warfarin in patients who underwent aortic bioprosthetic valve replacement. Our findings suggest that NOACs are noninferior to warfarin in terms of preventing thromboembolic events and major bleeding complications. No statistically significant differences were observed between the two treatment strategies, although a trend toward lower all-cause mortality and major bleeding with NOACs was noted, particularly in patients with lower bleeding risk profiles.

Despite these promising trends, the heterogeneity in the available evidence, including variations in study design and patient populations, warrants caution in interpreting the results. The data on secondary outcomes, such as valve thrombosis and minor bleeding, were limited, and no definitive conclusions could be drawn. The moderate risk of bias observed in several studies highlights the need for further RCTs to confirm these findings.

NOACs appear to be a viable alternative to warfarin for anticoagulation in patients following aortic bioprosthetic valve replacement. Given the lack of statistically significant differences and the potential advantages in terms of reduced monitoring, more large-scale trials are necessary to establish an optimal anticoagulation strategy for this patient population.

## Data Availability

No datasets were generated or analysed during the current study.
